# Platelet Derivatives and the Immunomodulation of Wound Healing

**DOI:** 10.3390/ijms23158370

**Published:** 2022-07-28

**Authors:** Fernanda Scopelliti, Caterina Cattani, Valentina Dimartino, Concetta Mirisola, Andrea Cavani

**Affiliations:** National Institute for Health, Migration and Poverty INMP/NIHMP, Via di S. Gallicano, 25, 00153 Rome, Italy; caterina.cattani@inmp.it (C.C.); valentina.dimartino@inmp.it (V.D.); concetta.mirisola@inmp.it (C.M.); andrea.cavani@inmp.it (A.C.)

**Keywords:** platelet, platelet derivatives, wound healing, immune system

## Abstract

Besides their primary role in hemostasis, platelets contain a plethora of immunomodulatory molecules that profoundly affect the entire process of wound repair. Therefore, platelet derivatives, such as platelet-rich plasma or platelet lysate, have been widely employed with promising results in the treatment of chronic wounds. Platelet derivatives provide growth factors, cytokines, and chemokines targeting resident and immigrated cells belonging to the innate and adaptive immune system. The recruitment and activation of neutrophils and macrophages is critical for pathogen clearance in the early phase of wound repair. The inflammatory response begins with the release of cytokines, such as TGF-β, aimed at damping excessive inflammation and promoting the regenerative phase of wound healing. Dysregulation of the immune system during the wound healing process leads to persistent inflammation and delayed healing, which ultimately result in chronic wound. In this review, we summarize the role of the different immune cells involved in wound healing, particularly emphasizing the function of platelet and platelet derivatives in orchestrating the immunological response.

## 1. Introduction

The skin has the largest surface area of all the organs of the human body and protects internal tissues from mechanical damage, pathogen entry, ultraviolet radiation, and extreme temperature. Therefore, it is frequently subject to injuries that require a rapid and co-ordinate cascade of events to re-establish the cutaneous integrity. Wound healing is the result of strict collaboration between resident and immigrated cells, innate and adaptive immune responses requiring a delicate equilibrium between protective and regulatory mechanisms [1,2]. Wound closure occurs in a temporary defined sequence that starts with hemostasis, followed by inflammatory, proliferative and, finally, remodeling phase. Dysfunctional cellular events or an abnormal production of cytokine and growth factors due to prolongation of the inflammatory phase can lead to chronic wounds and/or increased risk of infection [3,4].

Hemostasis initiates by platelets that aggregate upon exposure to subendothelial collagen, forming a plug that definitively blocks blood extravasation [5]. The conversion of prothrombin to thrombin is promoted by the coagulation cascade, which results from platelet activation, and has the ability to catalyze the conversion of fibrinogen to fibrin, leading to the formation of the thrombus.

Importantly, activated platelets expose a variety of membrane receptors and release soluble preformed mediators that regulate immune cell recruitment and activation [6]. Within 24–48 h, neutrophils and macrophages accumulate at the site of skin injury, initiating the inflammatory phase and providing protection against pathogens [5]. The proliferative phase encompasses processes that result in re-epithelialization, angiogenesis, collagen deposition, and formation of granulation tissues [7]. All these processes require a structural framework provided by fibroblasts responsible for the neo-synthesis of extracellular matrix (ECM) components. Under the stimulation of cytokines, such as interferon (IFN)-γ and transforming growth factor (TGF)-β, fibroblasts synthesize collagen and fibronectin to facilitate the closure of tissue gaps as well as restoration of mechanical strength [7]. Epidermal growth factor (EGF), fibroblast growth factor (FGF), and TGF-β promote re-epithelialization [8] through keratinocytes proliferation and migration over the wound bed. Simultaneously, angiogenesis is induced by various growth factors, such as vascular endothelial growth factor (VEGF), FGF, and platelet-derived growth factor (PDGF). Neo-angiogenesis is further facilitated through the release of proteolytic enzymes and metalloproteinases (MMP) by endothelial cells that dissolve basal lamina and surrounding tissue.

The present review aims to summarize and discuss the recent findings on the complex interaction between platelets and their derivatives used in clinical practice and the skin immune system during wound healing.

## 2. Innate and Adaptive Immune Cells in Wound Healing

The inflammatory phase starts within 24–48 h after injury and is characterized by the contribution of both resident and immigrated cells belonging to the innate and adaptive immune system [4].

The first wave of immigrated cells includes neutrophils and macrophages that are rapidly activated through interaction with resident cells, with platelets, or in response to pathogen-associated molecular patterns (PAMPs) and danger-associated molecular patterns (DAMPs). PAMPs and DAMPs activate molecular pathways upon the ligation of pattern recognition receptors (PRRs) expressed by responding cells, including Toll-like receptors (TLR), c-type lectin receptors, and nucleotide binding oligomerization domain (NOD)-Like Receptor [7]. Among skin resident cells, keratinocytes contribute substantially to the initiation of the inflammatory phase. They are equipped with TLRs, such as TLR-3, TLR-4, and TLR-9, whose expression is enhanced during acute wounds [8,9]. TLRs are triggered by their corresponding ligands, leading to the activation of downstream signaling molecules that induce the nuclear translocation of transcription factor NF-kB and/or activation of the mitogen-activated protein kinase (MAPK). The MAPK family includes p38 and Jun N-terminal kinase (JNK), whose activation leads to the transcription of target inflammatory cytokine genes such as IFN-1, tumor necrosis factor (TNF)-α, interleukin (IL)-8, IL-18 and IL-36γ, and chemokines (CCL20 and CCL27) [10,11]. Particularly, TLR-4 activates the TLR4/p38 and JNK MAPK signaling pathways, resulting in the stimulation of inflammatory cytokine production [9].

Neutrophils are the first circulating inflammatory cell to move to the site of the wound, with the primary role of protecting the injured skin from infections. Activated neutrophils produce antimicrobial peptides, proteases, and high concentrations of ROS that contribute to kill and degrade pathogens through the formation of neutrophil extracellular traps (NETs). NET formation starts with the activation of NADPH oxidase (NOX) complex through protein kinase C (PKC)-Raf/MERK/ERK, which in turn activates myeloperoxidase (MPO), neutrophil elastase (NE), and protein-arginine deiminase type 4 (PAD4) [12,13,14]. NETs contain chromatin filaments, histones, proteases, as well as granular and cytosolic proteins. Although NETs are important for pathogen clearance, their overexpression may be detrimental, since they prolong the inflammatory phase and delay wound healing. Besides, neutrophils have the ability to generate multiple cytokines and growth factors that contribute to the amplification of the inflammatory response to injury. At a later time point, neutrophils undergo apoptosis and are engulfed and cleared by macrophages [15]. Phagocytosis of apoptotic neutrophils results in the release of anti-inflammatory and reparative cytokines, in particular TGF-β and IL-10. Given the pronounced pro-inflammatory role of neutrophils in wound healing, animal models have shown that the depletion of neutrophils accelerates re-epithelialization [16]. In line with this finding, drugs that promote neutrophil apoptosis have a therapeutic potential to accelerate tissue repair [17].

Among immigrated cells, macrophages are the main orchestrators of the inflammatory phase of wound healing [18]. Blood monocytes rapidly accumulate at the site of skin injury and, under the influence of microenvironmental stimuli, polarize towards a pro-inflammatory phenotype, called M1. M1 secrete abundant production of protective cytokines, such as TNF-α, IL-6, IL-1β, IL-12, and IL-23, aimed at impeding pathogen entry and at alerting the adaptive immune system [19]. In addition, M1 macrophages are enriched in phagosomes containing ROS [20], and synthesize metalloproteinase (MMP), which digest ECM and thrombus for their migration. The digested ECM fragments act as immunostimulatory DAMPs, activating classical inflammation pathways through Toll-like receptor and inflammasome signaling [21,22]. Therefore, M1 macrophages dominate the early inflammatory phase of wound healing: they are involved in the protection of the injured skin from pathogens, removal of debrides and apoptotic neutrophils, and, finally, they function as antigen-presenting cells able to activate T lymphocytes thanks to the expression of MHC molecules and costimulatory molecules CD80 and CD86. Specific depletion of macrophages after excisional skin wounding would detrimentally affect healing by reducing the production of growth factors important in the repair process [23]. At later time point, macrophages convert into “alternatively activated macrophage”, an anti-inflammatory cell type known as M2. M2 macrophages control and resolve inflammation by releasing anti-inflammatory cytokines TGF-β and IL-10, which promote the production of ECM by fibroblasts, as well as neo-angiogenesis through the secretion of VEGF and PDGF [24,25,26,27]. On the contrary, M2 macrophages produce low levels of pro-inflammatory mediators, such as TNF-α, IL-12, and IL-8 [28]. Actually, the sharp distinction between M1 and M2 is artificial, since macrophages can differentiate into a multitude of phenotypes with specific function, depending on the microenvironment. In particular, at least 4 phenotypes of M2 macrophages have been described, termed a, b, c, d, and characterized by different expression of pro- and anti-inflammatory cytokines and with diverse pro-angiogenic capacity [29]. The M1 to M2 conversion is critical for the rapid repair of the wounded tissue. For example, in diabetic patients, the dysregulated M1 to M2 transition is responsible for delayed wound closure [30]. Once re-epithelialization occurs and the wound proceeds into the remodeling stage, a subset of macrophages in the wound regain their phagocytic phenotype and also acquire a “fibrolytic” profile, expressing the regulatory cytokine IL-10, metalloproteases, and arginase-1 [19]. Finally, macrophages could promote the transformation of fibroblasts into myofibroblasts by secreting TGF-β and PDGF-CC, both of which facilitate collagen deposition and scar formation [31,32,33]. Therefore, macrophages profoundly affect both the proliferative and the remodeling phases of wound repair by regulating the activity of fibroblasts. Macrophage overabundance have been associated with diseases characterized by fibroblast dysregulation, such as keloids and hypertrophic scars [5,34].

Recently, an atypical subtype of monocytes has been identified, termed segregated-nucleus-containing atypical monocytes (SatM), that share granulocyte characteristics, are regulated by CCAAT/enhancer binding protein β (C/EBPβ), and are responsible for scar formation. The atypical macrophages increase in numbers approximately 13 days after injury in mice [35].

The overwhelming presence of neutrophils and macrophages in wounds has potentially masked the importance of lymphoid cells in wound repair. However, recent studies have revealed the importance of both resident and immigrated T lymphocytes in wound healing. Human γδ T cells have been shown to promote wound healing among skin resident T cells, by releasing insulin-like growth factor-1 (IGF-1) [36]. Instead, the accumulation of cells of the adaptive immune system at the site of skin injury is delayed as compared with that of macrophages and neutrophils. Th22 cells promote keratinocyte proliferation and migration over the wound bed and favor collagen production by fibroblasts because of the production of the master cytokine IL-22 [37]. In a murine model of streptozotocin-induced type I diabetic mice, the impaired skin wound healing was almost completely restored with the administration of IL-22 [38]. Both resident and immigrated Foxp3^+^ T regulatory (Tregs) cells play an indispensable role in wound healing bysuppressing IFN-γ production through Th1 lymphocytes and decreasing the pro-inflammatory activity of macrophages [39]. Since the depletion of EGFR receptor reduces Treg infiltration in wounded skin and delays wound closure, regulatory activity appears strongly dependent on the expression of this receptor by Treg [39].

Dendritic cells (DCs) are specialized hematopoietic cells that serve as a bridge between innate and adaptive immunity [40,41]. DCs are categorized as conventional or myeloid DCs and plasmacytoid DCs, being the latter absent in steady state skin but rapidly recruited in inflamed skin. Moreover, Langerhans cells (LCs) are a specialized subset of dendritic cells (DCs) originating from immigrated monocytes that populate the basal and suprabasal layer of the epidermis.

Most of the data on the role of dendritic cells in wound healing derive from murine models [42,43]. The majority of LCs are lost immediately after the skin injury, but they are rapidly replaced by immigrated myeloid cells. Overall, DCs exert a pro-healing function in wound repair. In particular, LCs accumulation in the wound bed positively correlates with better outcome in diabetic wound [44]. Li and coll., using a CD11c-DTR mouse, showed that the deletion of CD11c+ DC upon injection of diphtheria toxin (DT) resulted in delayed wound closure [45].

Evidence concerning the role of dermal DC in wound closure has been demonstrated also by the crosstalk between epithelial cells and DCs residing in the corneal epithelium during corneal epithelial wound healing. The consequences of this interaction have been studied using B6-diphtheria toxin receptor transgenic mice (B6-DTR) depleted of their DC subtype: Gao et al. discovered that the lack of DCs in the cornea altered the epithelial response to injury, increased cell death, and impaired epithelial wound healing [46].

## 3. Platelet and the Immune Regulation of Wound Healing

With an average count of 150–450 × 10^9^ per liter, platelets together with erythrocytes are the most numerous cells in the blood circulation [47]. Platelets are primarily, but not exclusively, involved in the hemostasis phase of wound healing. A few seconds from injury, platelets adhere to the endothelial lesion via interaction of platelet glycoprotein Ib-IX-V-receptor (GPIb-IX-V) with collagen-bound von Willebrand factor (vWF) [48], and with the integrins αIIbβ3 and α2β1 that bind to fibrinogen, fibronectin, and collagen, respectively. The stable interaction provided by α2β-GPVI promotes platelet activation via an FcRγ-chain mediated mechanism, thus enabling the transition of platelet GPIIb/IIIa receptor from a low affinity into a high affinity state (“inside-out signaling”) [49,50]. In parallel, platelet aggregation is responsible for the initiation of signals aimed at recruiting and activating circulating immune cells. Indeed, platelets are equipped with several membrane receptors that sense endogenous and exogenous danger signals; they store in cytoplasmic granules a multitude of immunomodulatory substances, and, finally, they possess the capacity to synthesize newly formed cytokines, such as IL-1β, from pre-formed mRNA. Upon activation, molecules stored in platelet cytoplasmic granule are translocated to the cell membrane or released extracellularly. Platelets are equipped with three distinct types of cytoplasmic granules containing more than 300 molecules, including the VEGF, the epidermal growth factor (EGF), the FGF-2, IGF-1, chemokines (CXCL1, CXCL8, CCL3, CCL5, CCL7) and cytokines (IL-1, IL-6, TGF-β), which have fundamental roles in wound healing [6,51,52]. Furthermore, platelet recognize PAMPS thanks to the expression of cell-surface and intracellular TLR, namely TLR 2, 3, 4, 7, 9, and, at lower level, TLR-1 and 6 [53]. Engagement of TLR on platelets leads to selective and pleiotropic responses that have been only partially elucidated. TLR-2, TLR-4, and TLR-7 ligation strongly affects platelet-neutrophils interaction leading to neutrophil activation and NET formation [54,55]. Additionally, TLR-4 engagement by LPS has been reported to induce the platelet release of IL-1β, soluble CD40L and RANTES [56,57]. Platelets also contribute to pathogen clearance as a result of the release of anti-microbial peptides, such as thrombocidin-1, thrombocidin-2, β-defensin 1 and platelet microbicidal protein-1 (PMP-1) [58]. Upon platelet activation, P-selectin is rapidly exposed on the cell surface and mediates the interaction with P-selectin glycoprotein ligand 1 (PSGL-1) expressed by neutrophils and monocytes [59,60]. P-selectin-PSGL-1 ligation not only ensures adhesion between platelet and leukocytes, but also contributes to the activation of neutrophils and macrophages. In particular, platelet P-selectin acts in concert with platelet-derived RANTES so inducing the release of MCP-1 by monocytes, via nuclear translocation of NF-kB [61]. Platelet factor 4 (PF4) serves as a chemoattractant for monocytes and neutrophils in which enhances granule secretion in the presence of TNF-α [62]. Furthermore, PF4 inhibits macrophage apoptosis and promotes monocytes differentiation into macrophages [63]. MMP released by activated platelets can also regulate leukocytes migration, tissue degradation, and inflammation [64,65].

Upon platelet activation by thrombin, ADP or collagen, CD154 (CD40L) is stored in platelet α-granule and either translocated to the cellular membrane or released as soluble CD154 [66]. CD154, in its membrane-bound or soluble form, binds to CD40 expressed by a variety of immune cells, inducing the production of cytokines, chemokines, and other mediators of inflammation [67,68,69,70].

Schleicher et al. were able to demonstrate that activated platelets also expressed FAS ligand, an apoptosis inducing ligand primarily expressed by activated T cells, natural killer (NK) cells, and monocytes. Activated platelets as well as the isolated membrane fraction of activated platelets (but not of resting platelets) induced apoptosis in a dose-dependent manner in primary murine neuronal cells, human neuroblastoma cells, and mouse embryonic fibroblasts. Blocking of this platelet ligand or platelet depletion resulted in reduced apoptosis in models of retinal inflammation and stroke [71]. It was shown that upon activation, platelets synthesize and secrete IL-1β, a highly potent pro-inflammatory cytokine [72]. IL-1β upregulates both expression of adhesion receptors and secretion of IL-6 and IL-8 in endothelial cells and increases nitric oxide (NO) induced vascular permeability [73].

As the monocyte-macrophage system is the principal protagonist of both the inflammatory and regenerative phases of wound repair, platelet-monocyte interaction is extremely relevant in the context of wound healing. The interaction between platelet soluble and membrane-associated molecules on macrophages has variable effects in the context of wound tissues, depending on the phase of the repair process considered and on the experimental setting employed [74]. Activated platelets induce the nuclear translocation of NF-kB and expression of NF-kB-dependent inflammatory genes in monocytes [75]. IL-1β and PAF can act in concert with the engagement of PSGL-1 to amplify inflammatory gene expression, demonstrating signal integration and mechanisms for differential, time-dependent expression of key inflammatory products in a gene-specific fashion. Initial and subsequent studies demonstrated that monocyte chemotactic protein 1 (MCP-1), TNF-α, IL-8, and other inflammatory proteins are synthesized by monocytes upon P-selectin ligation of P-selectin glycoprotein ligand-1 (PSLG-1) on monocytes [61]. Platelet adhesion to monocytes also induces a CD14^+^CD16^+^ phenotype with enhanced proinflammatory properties [76]. The interaction of human macrophages with autologous platelets results in scavenger-receptor-mediated platelet uptake and enhancement of LPS-induced cytokines [77]. Moreover, GPIb-CD11b interaction was shown to polarize monocytes toward a proinflammatory phenotype and to promote inducible nitric oxide synthase–positive macrophage recruitment to the infected peritoneum [78].

Although a number of reports indicate that platelet interaction drives macrophages toward a M1 phenotype with prominent proinflammatory role [78], other data suggest that collagen-activated platelets exert anti-inflammatory functions by increasing the anti-inflammatory cytokine IL-10 and reducing TNF-α secretion by macrophages in a prostaglandin E2- or CD40L-dependent manner [79,80]. Recently, it has been demonstrated that platelet-derived nanovesicles activate IL-10 and TGF-β signaling in M1 macrophages, thus promoting their transition in M2 reparative macrophages [81]. Platelet ITAM receptor CLEC-2 is a key regulator of macrophage recruitment and activation. In fact, in a mouse model of acute respiratory distress syndrome, the CLEC-2 interaction with podoplanin on macrophages decreases their pro-inflammatory chemokine expression [82].

Accordingly, variable effects of platelets on DC have been reported on the basis of the type of DC investigated and on the experimental setting. Overall, DCs developed in the presence of thrombin-activated platelet release a decreased amount of IL-12p70 and TNF-α and increased production of immunosuppressive cytokine IL-10 [83,84]. As a consequence, DC exposed to platelets have impaired capacity to promote T cell proliferation and Th1 differentiation [84,85].

Intriguingly, the platelet interaction with neutrophils induces the exocytosis of neutrophil-derived extracellular vesicles (EV). EVs shuttle arachidonic acid into platelet intracellular compartments enriched in cyclooxygenase1 (Cox1), that process arachidonic acid to thromboxane A2 (TxA2). TXA2 released by platelets, in turn, induces endothelial cell expression of ICAM1, favoring further neutrophil recruitment [86].

Platelet exert variegate effects on T cell polarization. Due to the release of pleiotropic molecules, platelet may have opposite effects on T cell activation: PAF was shown to downregulate anti-CD3-induced T cells activation [87], and PF4 was shown either to promote Th1 and Th17 polarization [88], while RANTES and PDGF sustain T cell proliferation [89,90]. In addition, platelets enhance CD8+ T cell responses in a CD154-dependent manner in a murine model of wound healing [91]. Other reports demonstrated that platelet interaction with T cells decrease T cells proliferation and the release of IL-17 and type 1 cytokines [85,92,93]. Zhu et al. showed that platelets co-cultured with CD4^+^ T cells temporary enhanced both Th1 and Th17 differentiation followed by a more sustained suppression of Th1 and expansion of Foxp3+ Treg cells [94,95].

**Figure 1:**
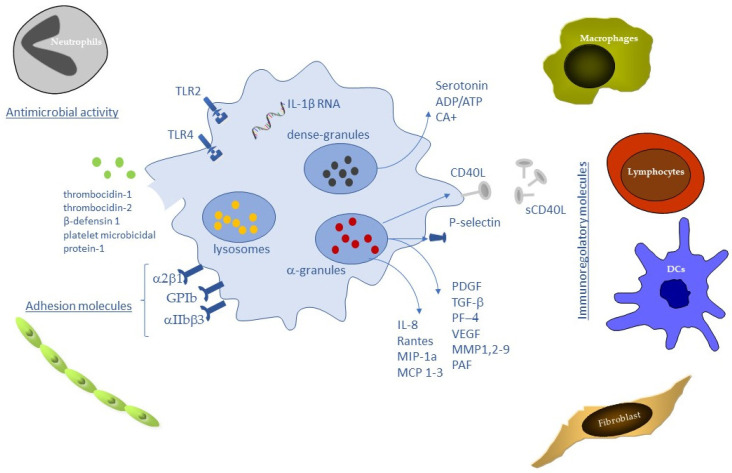
Platelet biomolecules during wound healing process.

It is pertinent to question how platelets are capable of modulating the balance between cell survival and apoptosis in tissues. SDF-1 acting with serotonin, ADP and *Sphingosine-1-phosphate* (S1P) favors cell survival. In contrast, a number of TNF-α related molecules including CD40L and soluble and membrane FAS ligand are secreted from platelets and promote apoptosis and dampen excessive inflammation [71]. Another platelet-expressed protein, TNF-related apoptosis-inducing ligand (TRAIL) regulates apoptosis in cells, including fibroblasts, smooth muscle cells, neutrophils, and monocytes [96]. Released ADP also favors platelet/leukocyte interactions [97] (Figure 1).

## 4. Platelet Derivates in Wound Healing

Over the last two decades, the administration of platelet lysate (PL), platelet-rich plasma (PRP), platelet gel (PG), or platelet rich fibrin (PRF), has gathered considerable attention for its potential use in the field of regenerative medicine as a therapeutic agent in a range of conditions, including chronic wounds. The curative properties of platelet derivates rely on the physiological reservoir of a variety of growth factors, cytokines and chemokines with potential pro-healing functions contained in platelet granule [98,99]. In particular, PRP or PL has been employed with variable results in order to hasten wound healing of diabetic and venous ulcers [100,101,102,103,104,105], enhance condrogenesis in osteoarthritis [106], prevent heterotopic ossification in hip arthroplasty [107], or affect wound healing [108]. Moreover, platelet-derived preparations might accelerate the regeneration of difficult-to-heal wounds by triggering an inflammatory cascade and playing an antimicrobial role [109,110]. Platelet-rich fibrin (PRF), a concentrate of cells and growth factors generated from the centrifugation of whole blood, is one local technique widely used in the treatment of periodontal defects and treatment of gingival recessions [108,111].

Platelet-based biomaterials can be prepared from a single donor (autologous or allogeneic) or from a pool of allogeneic donors. Unfortunately, there is a lack of standardized protocols for PRP preparation, and both in vitro data and the outcome of clinical application of platelet derived biomaterial vary depending on the type of procedures for PRP preparation, on the amount of anti-coagulant added to the blood sample, as well as on the time and modalities of storage, and on the type of substances added to induce platelet activation. The concentration of platelets in PRP after 2 density gradient centrifugation is 2–6 folds higher than that of whole blood. Platelet gel is obtained by addition of thrombin or collagen to lead to platelet activation in a network of fibrin. Recently, PRP has been administered in the form of hydrogels, based on polymers, such as chitosan, sponge-like dressings, or nano-microparticles to adsorb wound exudate and control the delivery of biomaterial. Alternatively, after being concentrated in PRP, platelets can be lysed by freeze/thaw cycles or ultrasound treatment, to obtain a solution of platelet lysate containing a cocktail of growth factors and cytokines.

**Figure 2:**
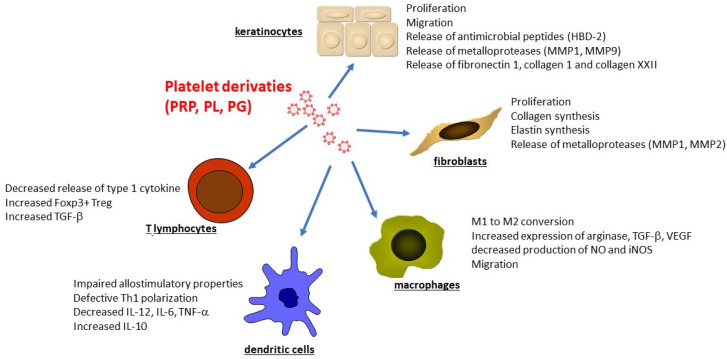
Effect of Platelet derivatives on human cells involved in Wound Healing.

There are valid expectations as regards the therapeutic benefits of PL on various cells belonging to the immune system. The in vitro effect of PL on endothelial cells, monocytes, fibroblasts and keratinocytes, has been investigated in terms of viability and proliferation, migration, angiogenesis, tissue repair pathway activation, and inflammatory response [112,113] (Figure 2). Human Keratinocytes or HACAT cell line exposed to 10% PRP exhibited increased proliferation and migratory properties as detected by a wound closure model, the scratch assay [19,114,115]. Whole transcriptome analysis of human keratinocytes treated by platelet derivatives, followed by ELISA and real-time PCR confirmation, revealed the induction of MMP9, fibronectin 1, collagen 1, and collagen XXII [116]. Accordingly, PRP induced the expression of MMP1 and MMP9 by HaCat cells [117]. Platelet lysate also increased the release of the antimicrobial peptide HBD-2 by human keratinocytes in a EGFR and IL-6-dependent manner [118]. At molecular levels, PL induced the viability, proliferation, and activation of important inflammatory pathways, such as ERK1/2 and NF-κB [112]. PL activation of ERK1/2 and NF-κB pathways has been demonstrated in two separate studies in keratinocytes. In particular, the study by El Backly and coworkers [109] showed that 5% PL, approximately corresponding to a physiologic platelet concentration in the pre-lysate platelet suspension, exerted the highest effect on wound closure, associated with activation of NF-κB. Both sub-physiologic (1%) and higher-than-physiologic (20%) concentrations resulted in a delaying effect, pointing to a dose-dependent effect of PL. Another study showed only ERK1/2 involvement with no variation of NF-κB phosphorylation in keratinocytes, whereas a slight but significant NF-κB inactivation was observed in fibroblasts [119,120].

Fibroblasts are equally affected by platelet-derived biomaterial. Exposure to PRP has been reported to increase the expression of type I collagen, elastin, MMP-1, and MMP-2 [121], as well as fibroblast proliferation [119,122,123]. Finally, Thrombin-activated PRP affects neo-angiogenesis, as demonstrated by the induction of the proliferation of HUVEC and endothelial cells [111,116,124].

Overall, in vitro data demonstrated an anti-inflammatory role of the platelet derivatives on both innate and adaptive immune cells, with slightly varied effects depending on the platelet preparate used.

Recent studies show that PG modulate the peripheral blood mononuclear cells (PBMC) production of several cytokines involved in tissue repair. PG induce a down-regulation of VEGF and b-FGF and an up-regulation of IL-10. Moreover, LPS treated PBMC decreased the release of IFN-γ and IL-12 when co-cultured with PG. Thus, PG treatment of inflammatory cells results in a downregulation of pro-inflammatory and pro-angiogenic cytokines, contributing to the establishment of a microenvironment more suitable for healing processes [125]. Our group demonstrated that PL dampens the macrophage secretion of pro-inflammatory cytokines and induces the release of arginase, TGF-β, and VEGF that may affect angiogenesis and tissue regeneration, thus facilitating the wound healing process [126]. Renn and coll. reported a decreased production of NO and iNOS by macrophage exposed to platelet derivatives and a decreased production of TNF-α when macrophages were treated with solvent/detergent-treated PL (SDPL) but not PL or PRP [127]. PL obtained by umbilical human cord blood induced a significant increase in monocytes migration with respect to serum-free conditions [128]. PRF are capable of reducing the LPS-induced inflammatory response of macrophages in particular by decreasing IL-1 and IL-6 and by increasing of arginase 1 expression [129]. In conclusion, PL can be an important contributor to macrophage polarization and monocyte migration during wound repair and support the employment of platelet-derived biomaterials in the treatment of chronic wounds.

A number of experimental groups have recognized PL as a valuable, non-animal alternative to the use of FBS in cell culture [110,130,131]. It is usually prepared from human donor platelets by apheresis products, which are treated by repeated freeze-thawing of platelet suspension to achieve the release of growth factors. After final centrifugation, which removes the debris, PL can be used as cell media supplement. Švajger et al. demonstrated the possibility of successfully differentiating DCs from monocytes using PL as an alternative serum supplement [132]. In comparison to AB serum or FBS, the use of PL allowed for optimal differentiation of DCs with characteristic phenotype and the capacity to respond to maturation stimuli. Date and coll. showed that IFN-γ-DC cultured in the presence of PL displayed an increased capacity to prime antigen-specific cytotoxic cells compared to controls [133]. However, discrepancies among the data reported in literature exist. Papait and coll. also demonstrated that PRP inhibits the differentiation of monocytes in DC obtained in vitro by cocktail of cytokines and induces CD163^+^CD206^+^ M2 macrophage with prominent regulatory properties thanks to the release of IL-10 and PGE2 [134]. Monocyte-derived DC cultured in 10% PL showed similar expression of co-stimulatory molecules compared to DC cultured in conventional serum-free medium, a more pronounced expression of CCR7 upon maturation, but displayed an impaired allostimulatory proprieties and defective induction of Th1 cells [135]. Platelets significantly inhibited the pro-inflammatory (IL-12, IL-6, TNF-α) and increased anti-inflammatory (IL-10) cytokine production of moDCs matured in presence of toll-like receptor (TLR)-dependent and TLR independent stimuli. Transwell assays and ultracentrifugation revealed that a soluble factor secreted by platelets, but not microvesicles, inhibited DC activation. Moreover, platelets and platelet-derived soluble mediators inhibited T cell priming and T helper differentiation towards an IFN-γ^+^ Th1 phenotype induced by moDCs. Overall, these results show that platelets are able to inhibit the proinflammatory properties of DCs, and may even induce an anti-inflammatory DC phenotype, with decreased T cell priming capacity.

We have recently demonstrated that PL has a dual effect on lymphocytes: PL treatment induces a transient increase of Th1 cytokines in the early phase, followed by an expansion of TGF-β^+^ T regulatory cells that promote tissue regeneration. The early effect on IFN-γ and TNF-α produced by T cells has two consequences: (i) the activation of resident cells, keratinocytes in particular, that become activated upon the influence of T cell derived stimuli and release a plethora of chemotactic factors for the recruitment of new waves of inflammatory cells; (ii) the promotion of innate immunity mechanisms aimed at preventing infections. However, at a later time point, PL treatment is responsible for a gradual decrease in the Th1 cytokines and, simultaneously, inducing the expansion of CD25^+^Foxp3^+^ T reg cells, releasing TGF-β [136].

Since platelets contain many pro-inflammatory molecules, and reduced platelet counts in patients or mice are linked with the host’s susceptibility to infections, it has been suggested that platelets protect the host from certain microbial infections and could restore the skin microbiome, which in turn can affect skin innate immune responses and promote normal wound repair.

In conclusion, the effect of PL on different cells involved in wound repair supports this application for this platelet derivative. Nevertheless, the rapid leakage and short half-life of growth factors limit PL clinical application. Moreover, a chronic wound environment can induce premature growth factor degradation and inactivation caused by elevated levels of matrix metalloproteinase activity. In order to overcome this problem, the incorporation of platelet derivatives in nanoparticles as potential wound dressing applications can be considered a promising approach aimed at preserving the bioactivity of the molecules released by activated platelets and to permit their prolonged release.

## Data Availability

Not applicable.
